# On the Chemical Discrimination of Vesical Calculi

**Published:** 1843-04

**Authors:** 


ART. IV.—*On the Chemical Discrimination of Vesical Calculi.* By E. SCHARLING, Prof. of Chemistry in the University of Copenhagen. Translated from the Latin, with an Appendix, by S. E. HOSKINS, M.D. With Plates.—*London*, 1842. 8vo, pp. 177.

IN our Number for April 1840 we gave a review of the original treatise of Dr. Scharling, and it was owing to our favorable judgment of it that Dr. Hoskins was led to undertake the translation now before us. In our former article we gave so full an account of the contents of Dr. Scharling's work, that we have only now to express our satisfaction that it has been translated, and that the task of translation has fallen into such able hands as those of Dr. Hoskins. The English version is at once faithful and elegant, the style having all the characters of an original composition. We have therefore no hesitation in saying that, with the excellent drawings of calculi with which it is illustrated, it is the most useful work on the subject, for a student or young practitioner, which we have in the English language.

